# The impact of a multifaceted intervention to reduce potentially inappropriate prescribing among discharged older adults: a before-and-after study

**DOI:** 10.1186/s40545-020-00236-0

**Published:** 2020-07-17

**Authors:** Muhammad Eid Akkawi, Mohamad Haniki Nik Mohamed, Mohd Aznan Md Aris

**Affiliations:** 1 Department of Pharmacy Practice, Faculty of Pharmacy, International Islamic University Malaysia, Kuantan, Malaysia; 2 Department of Family Medicine & Non-Communicable Disease Research Unit, Faculty of Medicine, International Islamic University Malaysia, Kuantan, Malaysia

**Keywords:** Potentially inappropriate medication, Potential prescribing omission, Older adults, Academic detailing, Smartphone app

## Abstract

**Background:**

Potentially inappropriate prescribing (PIP) is associated with the incidence of adverse drug reactions, drug-related hospitalization and other negative outcomes in older adults. After hospitalization, older adults might be discharged with several types of PIPs. Studies have found that the lack of healthcare professionals’ (HCPs) knowledge regarding PIP is one of the major contributing factors in this issue. The purpose of this study is to investigate the impact of a multifaceted intervention on physicians’ and clinical pharmacists’ behavior regarding potentially inappropriate medication (PIM) and potential prescribing omission (PPO) among hospitalized older adults.

**Methods:**

This is a before-and-after study that took place in a tertiary Malaysian hospital. Discharge medications of patients ≥65 years old were reviewed to identify PIMs/PPOs using version 2 of the STOPP/START criteria. The prevalence and pattern of PIM/PPO before and after the intervention were compared. The intervention targeted the physicians and clinical pharmacists and it consisted of academic detailing and a newly developed smartphone application (app).

**Results:**

The study involved 240 patients before (control group) and 240 patients after the intervention. The prevalence of PIM was 22% and 27% before and after the intervention, respectively (*P* = 0.213). The prevalence of PPO in the intervention group was significantly lower than that in the control group (42% Vs. 53.3%); *P* = 0.014. This difference remained statistically significant after controlling for other variables (*P* = 0.015). The intervention was effective in reducing the two most common PPOs; the omission of vitamin D supplements in patients with a history of falls (*P* = 0.001) and the omission of angiotensin converting enzyme inhibitor in patients with coronary artery disease (*P* = 0.03).

**Conclusions:**

The smartphone app coupled with academic detailing was effective in reducing the prevalence of PPO at discharge. However, it did not significantly affect the prevalence or pattern of PIM.

## Introduction

Older adults constitute the majority of medicine consumers as the number of diseases increases with age. This fact and other age-related physiological and pathological changes put older adults at higher risk to experiencing adverse drug reactions (ADRs), drug-drug interaction and drug-disease interactions compared to other age groups [1]. Potentially inappropriate prescribing (PIP) refers to the use of a medication whereby its risks outweigh its benefits or the underuse of a medication that has a valid indication with no contraindication in a particular patient [1]. Several previously published criteria addressed PIP facilitating healthcare professionals (HCPs) to appropriately prescribe medications for older adults. Beers [2] and the Screening Tool of Elderly Persons’ potentially inappropriate Prescription/ Screening Tool to Alert doctors to Right Treatment (STOPP/START) criteria [3] are amongst the most commonly used tools around the globe for this purpose. Beers and STOPP criteria address potentially inappropriate medications (PIMs), while START criteria discuss potential prescribing omissions (PPOs). The prevalence of PIP is quite high in older adults, and it is well established that PIP increases the incidence of ADRs and drug related hospitalization in this population [4, 5]. Hospital admissions should provide a good incentive for HCPs to optimize medications of hospitalized patients. However, PIP incidence is highly reported among discharged older adults [4, 6, 7] which may be attributed partially to the lack of HCPs’ knowledge regarding PIP in older adults [8, 9].

Educating HCPs is one of the approaches taken to improve the prescription quality and to reduce errors. A generally held belief is that a multifaceted intervention is much more effective than a single intervention in changing HCPs’ behavior [10]. A multifaceted intervention is simply defined as an intervention with more than one component [11]. Interventions with educational components were found to be among the most effective strategies implemented to change HCPs’ behavior [12]. Academic detailing, also known as educational outreach, refers to the personal visit of a trained person to HCPs at their surgeries to actively transfer information through presentations [12]. Academic detailing exhibited consistent positive effects on prescribing when it was applied as a single intervention or as a part of multifaceted interventions [13]. Another suggested key element for effective intervention is to provide the proposed recommendations at the point of care, where its application simplifies and eases clinical decision making [14]. A computerized decision support system (CDSS) is a software that analyzes data to help HCPs construct clinical decisions [15]. CDSS was implemented in different healthcare settings to improve prescribing appropriateness, ADRs and healthcare utilization while reducing prescription errors [16–18]. HCPs are increasingly using mobile computer devices (e.g. smartphones) in all healthcare settings which led to an accelerated expansion in the development of medical applications (apps) compatible with these devices [19]. This provides HCPs with easy access to vast and reliable resources at the point of care helping them make quick and informed clinical decisions. HCPs nowadays are more reliant on electronic resources. It was reported that HCPs spent double the time searching for information on electronic resources compared to printed resources [14]. The usage of medical apps at the point of care was associated with several positive outcomes from the HCPs’ and patients’ perspectives. It was reported that the use of mobile medical apps was more convenient for HCPs [19] and it improved their adherence to guidelines [20], accuracy, efficacy, productivity, and quality of the clinical decisions made [14, 21]. However, evidence is still limited, and more research is required to affirm these benefits [22, 23].

Interventions specifically targeted to reducing PIP in older adults rendered mixed results depending on the study design and setting as well as the type and intensity of the intervention [4, 17, 24, 25]. The objective of this study is to investigate the impact of academic detailing in conjunction with a smartphone app on the hospital HCPs’ behavior regarding PIP in older adults.

## Methods

### Study design and setting

This is a before-and-after study of a multifaceted intervention that was conducted in the multidisciplinary medical wards of a Malaysian tertiary hospital from April 2016 to October 2017. The study consisted of three phases. The first phase was a cross-sectional study that aimed to identify the prevalence and pattern of PIP among hospitalized older adults at discharge. This group of patients served as the control group. The second phase was the intervention phase; a multifaceted intervention for physicians and clinical pharmacists working in the medical wards. Two months after the end of the intervention, the third phase that took place was another cross-sectional study identical to the first phase study. The prevalence and pattern of PIP at discharge were reassessed on a different group of patients to be compared with the results found in the first phase study. The patients involved in the third phase study were considered as the intervention group.

### Study population and data collection

The targeted audience of the intervention were physicians and clinical pharmacists serving in the medical wards. To assess the impact of the intervention on their behavior on older adults, two groups of discharged older patients were recruited before and after the intervention. Patients ≥65 years of age who were discharged from the medical wards with at least one medication were involved in the study. Patients discharged to long-term care facilities were excluded. Eligible patients were conveniently selected using the discharge records from each ward. The collected data included the demographic details of the patients, vital signs, serum creatinine levels, abnormal laboratory results, comorbidities, past medical history, history of hospitalizations and the discharge medications. Combined preparation, which contains more than one active ingredient, was considered as one drug. The comorbidities were scored using the age-combined Charlson Comorbidity Index (AC-CCI) [26]. The discharge medications of the patients were reviewed twice to identify the PIMs and PPOs using version 2 of the STOPP/START criteria.

### Sample size

We calculated the sample size required for comparing two proportions (PIM prevalence at discharge for the control and intervention groups).

The following formula was used [27]:

$$ {n}=\left(\frac{\text{Pc}\left(1-\text{Pc}\right)}{k}+ \text{Pi}\ \left(1-\text{Pi}\right)\right)\ {\left[\left(\text{Z}_{1-\alpha}+\text{Z}_{1-\beta}\right)/\left(\text{Pc-Pi}\right)\right]}^2. $$

where,

n: the sample size for each study group.

Pc: the proportion of the event (presence of PIM) in the control group.

Pi: the proportion of the event (presence of PIM) in the intervention group.

*k*: Ratio between the sample sizes of the two groups.

Z_1-α_: Z statistic for a level of confidence (one side).

Z_1-β_: Z statistic for a level of the power (80%).

$$ \mathrm{n}=\left(\frac{0.3\ \left(1-0.3\right)}{1}+\kern0.37em 0.2\ \left(1-0.2\right)\right)\ {\left[\left(1.64+0.84\right)/\left(0.3-0.2\right)\right]}^2=229. $$

Therefore, to test the difference in the proportions between the two groups, each group should involve at least 229 patients.

### The intervention

#### Designing the intervention framework

The intervention aimed to change the HCPs’ behavior regarding prescribing for older adults. Therefore, it was designed using the “capability, opportunity and motivation” behavior (COM-B) model [28]. The targeted behavior was prescribing medications for older adults. The details of the targeted behavior were specified. See Table 1.

**Table 1:** Specifying the target behavior

Target behavior	Prescribing medications for hospitalized older adults
**Who needs to perform the behavior?**	Physicians and hospital pharmacists.
**What do they need to do differently to achieve the desired change?**	Follow the criteria for prescribing in older adults. Check the appropriateness of each medication intended to be prescribed for an older adult.
**When do they need to do it?**	During writing prescriptions and reviewing medications of older adults.
**Where do they need to do it?**	At the point of care.
**How often do they need to do it?**	Every time they prescribe and review medications for older adults.
**With whom do they need to do it?**	Alone, or in collaboration with other colleagues.

**Fig. 1:**
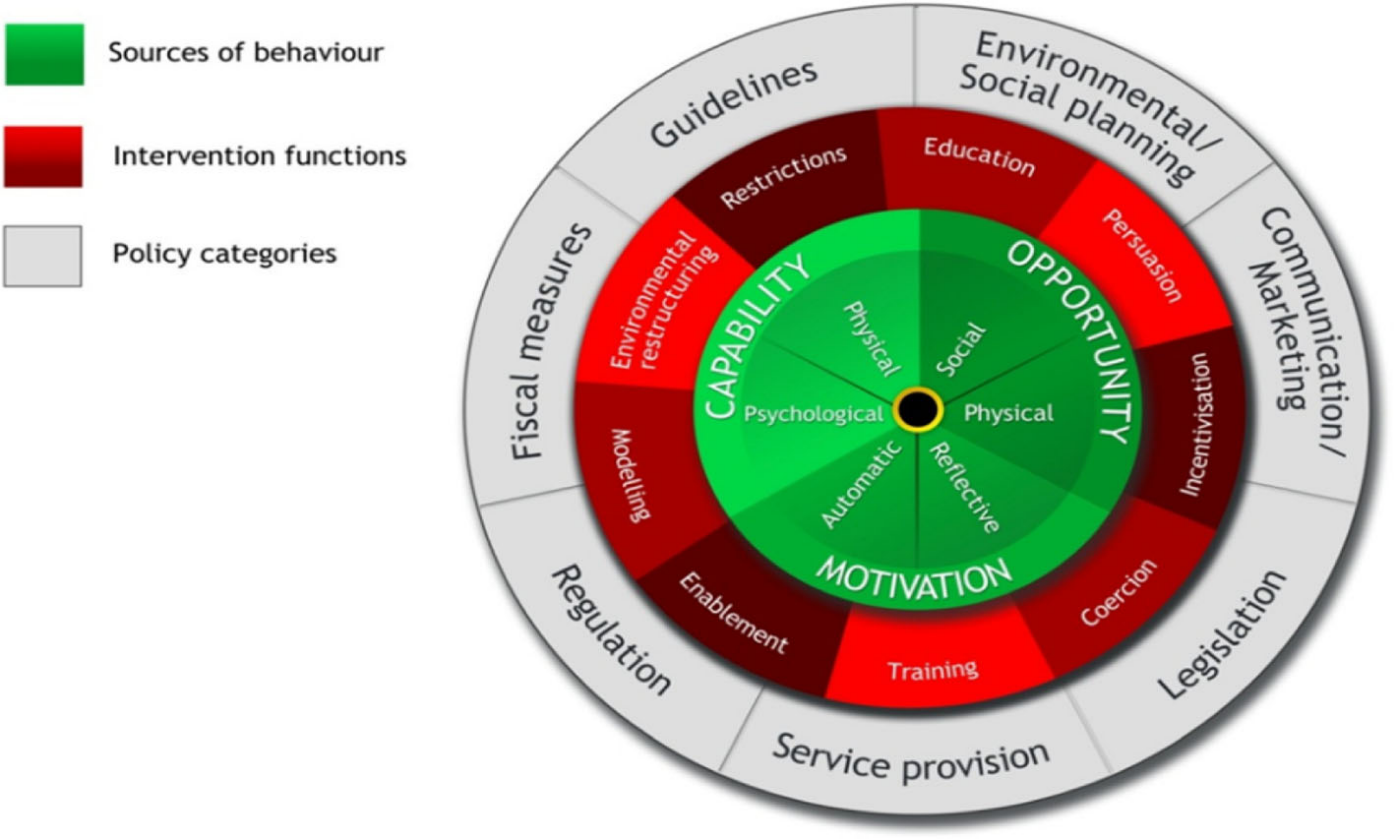
The behavior change wheel. Used with a permission from [28]

Based on the results of the first phase study, a behavioral analysis was conducted to identify COM-B components that needed to be changed. It was found that psychological capability, physical opportunity, reflective and automatic motivation of the HCPs needed to evolve in order to change the targeted behavior. See Fig. 1. Using the COM-B matrix, intervention functions were specified and thus a multifaceted intervention -consisting of academic detailing and provision of guidelines at the point of care- was believed to be effective in achieving the goal of the study.

#### The components of the intervention

The intervention consisted of three face-to-face sessions of academic detailing coupled with an introduction of a new smartphone app. The targeted HCPs for this intervention were physicians and clinical pharmacists serving at the medical wards of the hospital. The first session was about potentially inappropriate prescribing and why older adults are much more vulnerable to this problem. In addition, the session discussed explicit criteria used for appropriate prescribing in older adults, particularly the STOPP/START criteria, and the results of comparable studies on hospitalized patients. The second session was a briefing about the newly developed smartphone app; how to use it and its advantages. Then, the participants were asked to answer -using the smartphone app- six clinical vignettes that address the treatment of common diseases in older adults. Consecutively, the answers of the clinical vignettes were discussed with the participants as a form of training on using the smartphone app.

Three months later, another academic detailing session was delivered to the clinical pharmacists. The goal of this session was to revise the information delivered during the first two sessions and to emphasize on the use of the developed smartphone app. Moreover, the participants were informed about the most frequent PIPs encountered in the first phase study. To avoid Hawthorn’s effect, the HCPs were not aware that the discharge medications of their patients would be analyzed again to identify PIPs.

**Fig. 2:**
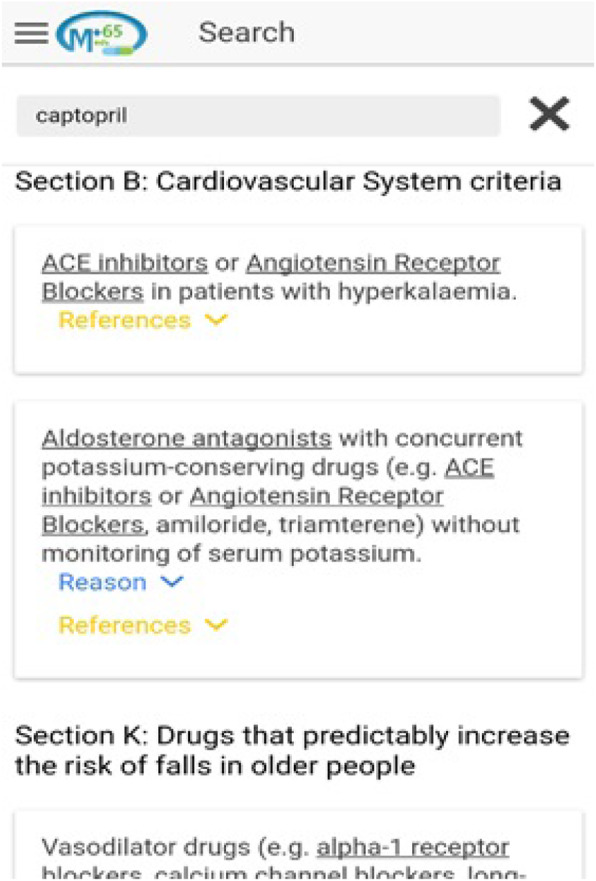
Screenshot shows searching for captopril-related recommendations on Plus65 Med^©^

A new smartphone app was created based on the STOPP/START criteria version 2 by using JavaScript programming language. The criteria were converted into a smartphone app, named “Plus65 Med^©^”. Some additional features were added to the app to ease the use of the criteria. The STOPP/START criteria use the names of drug classes for their recommendations. However, in the Plus65 Med^©^, the individual drug names were added, and they were made searchable. For example, if a user types “captopril” (an angiotensin converting enzyme (ACE) inhibitor) in the search bar, all recommendations under STOPP criteria and related to ACE inhibitors will appear. See Fig. 2. Also, it can be searched using disease names or drug class names to find the relevant recommendations. Plus65 Med^©^ was made available on Google Play and App. Store for free. The participants were asked to use Plus65 Med^©^ later during their daily practice and to provide feedback.

### Statistical analysis

Mean (SD) and median (IQR) were used to describe patients’ features and results. The data was analyzed using the Statistical Package for the Social Sciences version 24.0 (IBM SPSS Statistics 24). Shapiro-Wilk normality test was conducted to test the normality of continuous variables and to select the statistical tests subsequently. Pearson’s chi-squared test was used to compare the distribution of categorical variables between the control and intervention groups. Mann-Whitney *U*-test was used to compare the nonparametric variables between the two groups. A binominal logistic regression model was applied to test the impact of the intervention on PPO while controlling other variables that may affect the prevalence of PPOs. The variables included in the model were patient’s group (intervention vs control), sex, race and number of discharge medications, AC-CCI and the presence of PIM at discharge. Race categories were treated as dummy variables with Malay being the reference race. For sex, male was considered as the reference sex. Chi square test was used to evaluate the significance of the model. Nagelkerke R^2^ value was used to calculate the explained variation of the dependent variable (PPO). Significance was determined as 5%.

## Results

### Characteristics of the study population

Each group in the study consisted of 240 patients. The median age of the control group and intervention group was 70 and 72 years, respectively (*P* = 0.017). The number of female patients was higher in the intervention group as compared to the control group (*P* = 0.003). The other characteristics were not significantly different between the two groups. See Table 2**.**

**Table 2:** Characteristics of the study population (*N*: 480)

Variable	Control group (***N***:240)	Intervention group (***N***:240)
Age (year)		
Mean (SD)	71.9 (5.8)	72.9 (5.7)
Median (IQR)	70 (67–75)	72 (68–81)
Sex		
Male [*N* (%)]	141 (58.8)	108 (45)
Female [*N* (%)]	99 (41.3)	132 (55)
Race		
Malay [*N* (%)]	180 (75)	171 (71.3)
Chinese [*N* (%)]	46 (19.2)	54 (22.5)
Indian [*N* (%)]	14 (5.8)	15 (6.2)
AC-CCI		
Mean (SD)	4.6 (1.6)	4.83 (1.7)
Median (IQR)	4 (3–5)	4 (4–6)
Discharge medications		
Mean (SD)	5.9 (2.5)	6.2 (2.5)
Median (IQR)	6 (4–7)	6 (5–8)
History of hospitalization (during the last 12 months)		
Yes [*N* (%)]	88 (36.7)	76 (31.7)
No [*N* (%)]	152 (63.3)	164 (68.3)

*SD* standard deviation, *IQR* interquartile range, *AC-CCI* age combined Charlson comorbidity index.

### Impact of the intervention on potentially inappropriate prescribing

**Fig. 3:**
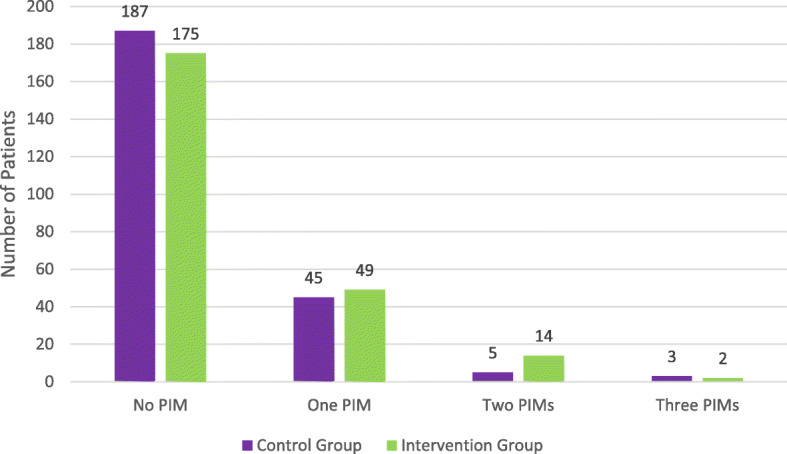
Distribution of PIMs among the two groups

At discharge, the prevalence of PIM was lower in the intervention group (22%) than that of the control group (27%). However, this difference was not statistically significant (*P* = 0.245). In addition, no significant difference was found in terms of number of PIMs per patient between the control and intervention groups (*p* = 0.172). See Fig. 3.

The most common PIMs in both groups were prescribing vasodilators, that increases the risk of falls, and were used among patients with persistent postural hypotension. The number of encountered STOPP criteria was 15 out of a possible 80 (18.75%) criteria in the control group as compared to 10 (12.5%) in the intervention group. Nevertheless, no significant differences were found in regards to individual STOPP criteria between intervention and control groups. See Table 3.

**Table 3:** Comparison of prevalence rates of PIM among the control and intervention groups

Type of PIMs according to STOPP criteria	Control group ***N*** (%)	Intervention group ***N*** (%)
**Drug Indication Criteria**		
“Any duplicate drug class prescription”	2 (0.8)	0
**Coagulation System**		
“Aspirin plus clopidogrel as secondary stroke prevention, unless the patient has a coronary stent(s) inserted in the previous 12 months or concurrent acute coronary syndrome or has a high grade symptomatic carotid arterial stenosis”	3 (1.3)	0
“Aspirin in combination with vitamin K antagonist, direct thrombin inhibitor or factor Xa inhibitors in patients with chronic atrial fibrillation”	1 (0.4)	0
“Ticlopidine in any circumstances”	5 (2.1)	7 (2.9)
**Central Nervous System and Psychotropic Drugs**		
“First-generation antihistamines”	1 (0.4)	1 (0.4)
**Renal System**		
“NSAID’s if eGFR < 50 ml/min/1.73m^2^”	1 (0.4)	1 (0.4)
“Metformin if eGFR < 30 ml/min/1.73m^2^”	6 (2.5)	4 (1.7)
**Gastrointestinal System**		
“Drugs likely to cause constipation in patients with chronic constipation where non-constipating alternatives are available”	1 (0.4)	0
“Proton pump inhibitor for uncomplicated peptic ulcer disease or erosive peptic oesophagitis at full therapeutic dosage for > 8 weeks”	14 (5.8)	13 (5.4)
**Respiratory System**		
“Anti-muscarinic bronchodilators with a history of narrow-angle glaucoma or bladder outflow”	2 (0.8)	0
**Musculoskeletal System**		
“Long-term NSAID or colchicine (> 3 months) for chronic treatment of gout where there is no contraindication to a xanthine-oxidase inhibitor”	2 (0.8)	1 (0.4)
**Endocrine System**		
“Sulphonylureas with a long duration of action with type 2 diabetes mellitus”	1 (0.4)	0
“Beta-blockers in diabetes mellitus with frequent hypoglycaemic episodes”	10 (4.2)	4 (1.7)
**Drugs that predictably increase the risk of falls in older people**		
“Vasodilator drugs with persistent postural hypotension”	23 (9.6)	24 (10%)
**Analgesic Drugs**		
“Use of regular opioids without concomitant laxative”	0	4 (1.7)

**Fig. 4:**
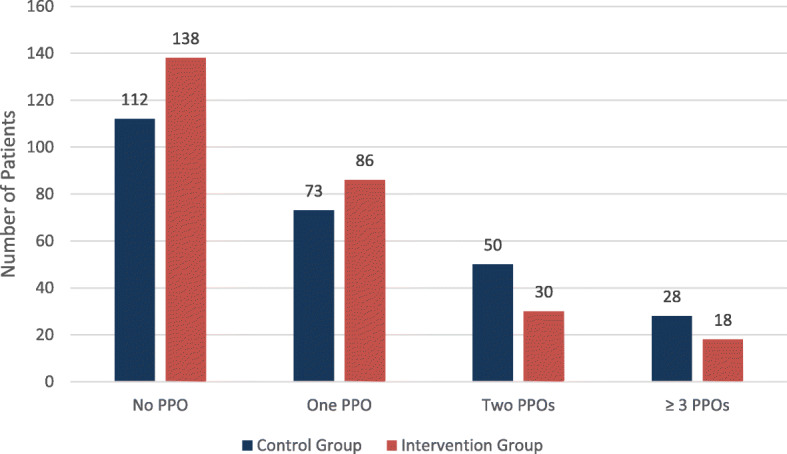
Distribution of PPOs among the study group

The prevalence of PPO in the intervention group was significantly lesser than that of the control group (42% Vs. 53.3%); *P* = 0.014. Additionally, the median number of PPOs per patient in the intervention group was significantly low compared to patients in the control group (*P* = 0.017). Forty-eight patients in the intervention group were discharged with more than one PPO, whereas the number was significantly higher in the control group (78 patients); *P* = 0.002. See Fig. 4.

The two most common PPOs in the control group were the omission of vitamin D supplements among patients with a history of falls and the omission of ACE inhibitor in patients with documented coronary artery disease (CAD). However, the prevalence rates of these two PPOs were reduced significantly in the intervention group. See Table 4.

**Table 4:** Comparison of the prevalence rate of PPO among the control and intervention groups

Type of PPOs according to START criteria	Control Group ***N*** (%)	Intervention group ***N*** (%)
**Cardiovascular System**		
“Vitamin K antagonists or direct thrombin inhibitors or factor Xa inhibitors in the presence of chronic atrial fibrillation”	2 (0.8)	7 (2.9)
“Antiplatelet therapy with a documented history of coronary, cerebral or peripheral vascular disease”	10 (4.2)	5 (2.1)
“Statin therapy with a documented history of coronary, cerebral or peripheral vascular disease”	11 (4.6)	11 (4.6)
“Angiotensin Converting Enzyme (ACE) inhibitor with systolic heart failure and/or documented coronary artery disease”	30 (12.5)*	16 (6.7)*
“Beta-blocker with ischaemic heart disease”	22 (9.2)	16 (6.7)
“Appropriate beta-blocker (bisoprolol, nebivolol, metoprolol or carvedilol) with stable systolic heart failure”	6 (2.5)	6 (2.5)
**Respiratory System**		
“Regular inhaled ß2 agonist or antimuscarinic bronchodilator mild to moderate asthma or COPD”	11 (4.6)	17 (7.1)
“Regular inhaled corticosteroid for moderate-severe asthma or COPD”	10 (4.2)	6 (2.5)
**Musculoskeletal System**		
“Vitamin D supplements in older people who are housebound or experiencing falls or with osteopenia”	37 (15.4)**	14 (5.8)**
“Xanthine-oxidase inhibitors with a history of recurrent episodes of gout”	2 (0.8)	3 (1.3)
**Endocrine System**		
“ACE inhibitor or Angiotensin Receptor Blocker (if intolerant of ACE inhibitor) in diabetes with evidence of renal disease”	20 (8.3)	29 (12.1)
**Urogenital System**		
“Alpha-1 receptor blocker with symptomatic prostatism, where prostatectomy is not considered necessary”	9 (3.8)	6 (2.5)
“5-alpha-reductase inhibitor with symptomatic prostatism, where prostatectomy is not considered necessary”	12 (5.0)	9 (3.8)

*COPD* chronic obstructive pulmonary disease.

*p = 0.03; **p = 0.001

The applied logistic regression model statistically and significantly predicted discharging with PPO, χ^2^(7) = 13.68, *P* = 0.006. The model explained 37.5% (Nagelkerke R^2^) of the variance in PPO and correctly classified 70% of cases. Furthermore, the applied logistic regression revealed that the control group was significantly more likely to discharge with PPO compared to the intervention group after controlling for the other variables (adjusted odds ratio = 1.59; 95% CI 1.1–2.3; *P* = 0.015).

## Discussion

This study investigated the impact of a multifaceted intervention on the behavior of physicians and clinical pharmacists in regards to potentially inappropriate prescribing. The study compared the prevalence and pattern of discharge PIPs before and after the intervention. Before the intervention, the prevalence of PIMs at discharge was found to be 27% which is lower than what was found elsewhere [4, 29, 30]. Consistent with other studies, the most common PIM was related to medications that increases the risk of falls in older adults [31–35]. In terms of PPO, the prevalence was 53.3% which is relatively higher than that reported from other Asian studies [36, 37]. The omission of vitamin D supplement in patients with a history of falls or fractures was the most encountered PPO. This finding is in agreement with reports from other studies [6, 33, 34].

This study also showed an insignificant difference in the prevalence and number of PIMs between the intervention and control groups. The study applied two interventional components, namely academic detailing and a new smartphone app targeting HCPs. Medical literature revealed that academic detailing has a grander impact on HCPs’ behavior compared to the distribution of printed materials, audit, feedback or opinion leaders [12]. In fact, a majority of the published interventional studies that were conducted to reduce PIP took place in primary care or nursing homes [24, 38–42]. A few studies were conducted in hospital settings. Najjar et al. used educational sessions paired with printed materials and combined with a clinical pharmacist’s intervention to reduce the prescription of PIMs during hospitalization [43]. The authors disclosed that the intervention significantly reduced the incidence rate of PIMs from 61 to 29.5%. The disagreement between the results of that study and those of this research may be due to the components of the intervention and tools used to identify PIMs. The researchers in the above-mentioned study used both Beers criteria and the STOPP criteria to detect PIMs, which gave high incidence of PIM (61%) as compared to this research (27%). In addition, the researchers investigated the medication during hospitalization but not discharge medications. More importantly, Najjar et al. involved -in addition to the educational intervention- a clinical pharmacist’s intervention where the clinical pharmacist reviewed the medications and gave recommendations to the physicians who accepted 61.83% of the proposed recommendations. Similarly, the review of medications by a medical interest was effective in reducing PIMs of discharged older patients in Spain [44].

The innovative aspect of our study is the developed smartphone app that was based on the STOPP/START criteria. To our knowledge, this is the first study that applied handheld devices to reduce inappropriate prescribing in older adults. However, involving CDSS as part of the intervention to reduce PIP in hospital settings was reported in the literature. The studies showed that CDSS is effective in reducing the PIMs among older inpatients [45–50]. These studies contradicted what has been found in our study, where the intervention failed to reduce PIM as compared with the control group significantly. These differences may be attributed to several reasons. Unlike using CDSS in the studies mentioned above, the use of Plus65 Med^©^ was not compelling when prescribing a medication. The use of the app was self-reported by the HCPs, and we did not have a mechanism to make sure that it was being used at the point of care. A computerized alert system was not applicable to the study site as the hospital dispensing system was not fully computerized, and that was what inspired the development of this smartphone app. Another possible reason for the differences is the applied PIM explicit criteria. Most of the studies investigated the impact of intervention on selected PIMs or diseases, which makes the comparison between different studies inaccurate, unless it takes into consideration the type and number of the studied PIPs. For instance, Terrel et al. chose to study the impact of the CDSS on reducing the prescriptions of only nine high-use medications from the Beers list [49] and Mattison et al. designed a CDSS that alerted physicians when they intend to prescribe 16 medications from the Beers list [46]. Other reported studies focused on specific drug classes. Agostini et al. restricted their study to four sedative-hypnotic agents that are inappropriately used among older adults [47]. Likewise, Peterson et al. targeted to correct the inappropriate use of 12 psychotropic agents in their first study [48] whereas, the researchers limited their second study to medications with high-anticholinergic activity [45]. In contrary to the above-mentioned studies, this study used the full list of medication classes stated in the STOPP criteria.

In contrast to the findings pertaining to PIMs, the current study found that the prevalence and number of PPOs were significantly lower in the intervention group patients. Even after controlling the baseline differences between the two groups, the prevalence of PPOs was still significantly lower in the intervention group. This can be explained by the fact that HCPs before intervention tended to deprescribe inappropriate medications rather than prescribe new medications that were inappropriately omitted. In the first phase of the current study, it was found that HCPs were able to significantly reduce the prevalence of admission PIMs but not PPOs [51]. After the intervention, HCPs may become more aware of PPO than previously, but the intervention failed to further enhance their role in reducing PIMs. The intervention was effective in reducing the prevalence of the two most common PPOs encountered in the patients; the omission of vitamin D in patients with a history of falls and the omission of ACE inhibitors in patients with CAD. During the academic detailing, the main results of the first phase study were presented, which might draw the HCPs’ attention to the underprescription of these medications. However, no significant changes were found in other PPOs. This might be related to the degree of HCPs’ agreement on a particular recommendation. For example, it was noticed that most of the HCPs prefer not to prescribe an ACE inhibitor for a patient with any degree of chronic kidney disease. Hence, it was not surprising not to see a significant reduction in the omission of ACE inhibitor in patients with DM and renal failure, although it was a common PPO. It was also found that only single study addressed the impact of an intervention for HCPs on PPOs in hospitalized older adults, where the multifaceted intervention was not effective in reducing PPOs as measured by the modified ACOVE criteria [50]. This disagreement may be attributed to different criteria used, as the researchers of that study used implicit criteria. The current intervention would theoretically have a significant clinical impact on the patients by reducing the incidence of new fractures and by preventing worsening of heart failure condition or recurrence of CADs. To ascertain this would happen, a larger randomized study investigating the clinical outcomes by long-term follow-up should be considered in the future.

### Strengths and limitations

This study is believed to be the first that provides PIP guidelines to HCPs at the point of care through a smartphone app. In addition, it is the first interventional study to reduce PIP in Malaysia. Moreover, the study involved a representative number of patients. On top of that, the intervention was designed using a well-established behavioral model. However, this study has several limitations. It took place in one hospital only, and since there were no other similar studies in Malaysia to compare it with, it could not be assumed that the findings represent the practice generally in all Malaysian hospitals. Besides, the endpoint of the study was the presence of PIP with no follow up to trace the negative outcomes of the presenting PIPs. Lastly, the use of the introduced smartphone app was self-reported, with no method to confirm its usage at the point of care, and that might affect the internal validity of the intervention.

## Conclusions

This study showed that a multifaceted intervention involving academic detailing and a smartphone app was effective in reducing the prevalence and number of discharge PPOs per patient among older adults. The omission of vitamin D in patients with a history of falls, and the omission of ACE inhibitor in patients with a history of CAD were significantly reduced at discharge after the intervention. On the other hand, this intervention did not significantly change the incidence of PIM among the discharged patients. The HCPs’ agreement on the used PIM criteria and the possible underuse of the smartphone app may attribute to this outcome.

## Data Availability

The datasets used and/or analyzed during the current study are available from the corresponding author on reasonable request.
